# In Vitro Phytochemical Screening, Cytotoxicity Studies of *Curcuma longa* Extracts with Isolation and Characterisation of Their Isolated Compounds

**DOI:** 10.3390/molecules26247509

**Published:** 2021-12-11

**Authors:** Madhuri Grover, Tapan Behl, Aayush Sehgal, Sukhbir Singh, Neelam Sharma, Tarun Virmani, Mahesh Rachamalla, Abdullah Farasani, Sridevi Chigurupati, Amal M. Alsubayiel, Shatha Ghazi Felemban, Mohit Sanduja, Simona Bungau

**Affiliations:** 1 School of Pharmaceutical Sciences, MVN University, Palwal 121102, India; grovermadhuri@gmail.com (M.G.); tarun.virmani@mvn.edu.in (T.V.); mohitsanduja007@gmail.com (M.S.); 2 Chitkara College of Pharmacy, Chitkara University, Rajoura 140401, India; aayushsehgal00@gmail.com (A.S.); sukhbir.singh@chitkara.edu.in (S.S.); neelam.mdu@gmail.com (N.S.); 3 Department of Biology, University of Saskatchewan, 112 Science Place, Saskatoon, SK S7N 5E2, Canada; maheshgupta65@gmail.com; 4 Biomedical Research Unit, Medical Research Centre, Jazan University, Jazan 45142, Saudi Arabia; aofarasani@jazan.edu.sa; 5 Department of Medical Laboratory Technology, College of Applied Medical Sciences, Jazan University, Jazan 45142, Saudi Arabia; 6 Department of Medicinal Chemistry and Pharmacognosy, College of Pharmacy, Qassim University, Buraydah 52571, Saudi Arabia; drsridevich@gmail.com; 7 Department of Pharmaceutics, College of Pharmacy, Qassim University, Buraydah 52571, Saudi Arabia; asbiel@qu.edu.sa; 8 Department of Medical Laboratory Science, Fakeeh College for Medical Sciences, Jeddah 21461, Saudi Arabia; sfelemban@fcms.edu.sa; 9 Department of Pharmacy, Faculty of Medicine and Pharmacy, University of Oradea, 410073 Oradea, Romania

**Keywords:** *Curcuma longa*, bisdemethoxycurcumin, cancer, cancer cell lines, curcuminoids, ethanolic extract

## Abstract

The *Curcuma longa* plant is endowed with multiple traditional and therapeutic utilities and is here explored for its phytochemical constituents and cytotoxic potential. Turmeric rhizomes were extracted from three different solvents and screened for the presence of different phytochemical constituents, observation of which indicated that the polar solvents favoured extraction of greater versatile phytochemical constituents. These extracts were investigated for their cytotoxic potential by MTT (3-(4,5-dimethylthiazol-2-yl)-2,5-diphenyltetrazolium bromide) assay on three different of cell lines including SCC-29B (oral cancer cell line), DU-145 (prostate cancer cell line) and the Vero cell line (healthy cell line/non-cancerous cell line). This assay was performed by taking three extracts from isolated curcuminoids and a pure bioactive compound bisdemethoxycurcumin (BD). Bisdemethoxycurcumin was isolated from curcuminoids and purified by column and thin-layer chromatography, and its structural characterisation was performed with different spectroscopic techniques such as FTIR, NMR (^1^H Proton and ^13^C Carbon-NMR) and LC-MS. Amongst the extracts, the ethanolic extracts exhibited stronger cytotoxic potential against the oral cancer cell line (SCC-29B) with an IC_50_value of 11.27 μg/mL, and that this was too low of a cytotoxicity against the Vero cell line. Although, curcuminoids have also shown a comparable cytotoxic potential against SCC-29B (IC_50_ value 16.79 μg/mL), it was not as potent against the ethanolic extract, and it was even found to be cytotoxic against healthy cell lines at a very low dose. While considering the isolated compound, bisdemethoxycurcumin, it also possessed a cytotoxic potential against the prostate cancer cell line (DU-145) (IC_50_ value of 93.28 μg/mL), but was quite safe for the healthy cell line in comparison to doxorubicin.

## 1. Introduction

Cancer is a chronic inflammatory-linked disorder and the second leading cause of death worldwide. Despite great advancement in its diagnosis and therapeutic measures [1,2,3,4], this disease is spreading at a very high pace, and it is estimated to reach 21 million by the year 2030 [5,6]. According to the World Health Organization (WHO) and the latest global cancer data released by the International Agency for Research on Cancer (IARC), approximately 851,678 mortality and 1,324,413 new cancer cases were reported in India in 2020. Amongst the latest cases, around 0.64 million were men with the highest fraction of oral (10.3%), lung (5.5%) and stomach (4.5%) cancer types, while women were reported with breast (13.5%), cervix (9.4%) and ovary (3.5%) cancer as the top three cancers amongst the total 0.67 million cases [7].

The rapid prevalence of the disease creates much of the financial and psychological burden on people living in developed and developing countries, thereby constantly increasing the demand for a more economical, targeted and effective approach. Although many therapeutic approaches are available, such as surgery, radiotherapy, chemotherapy, immunotherapy, cancer vaccination, photodynamic therapy, stem cell therapy or their combination, all these are associated with many limitations such astoxic reactions [8], low bioavailability, non-targeted or monotargeted action and rapid clearance [9,10,11]. Hence, scientists are more inclined towards medicinal plants or their phytochemicals, which can serve as a promising future aspect. Furthermore, many scientific reports have confirmed that natural dietary agents, such as vegetables, fruits, spices and phytochemicals, possess a strong antiproliferative [12,13] and cytotoxic potential [14]. For instance, several phytochemicals, such as curcumin, epigallocatechin, isothiocyanates and garcinol, are under clinical trials and can soon hit the market [15].

*Curcuma longa,* commonly called turmeric (family Zingiberaceae), is mostly grown in tropical and subtropical regions worldwide [16]. It is used extensively as an Indian food component for its flavouring and colouring properties and also traditionally for treating many ailments. It owns versatile biological activities such as scavenging free radicals [17], protects from protozoal diseases [18,19,20], reduces the toxicity of snake venom [20,21], anti-microbial [22], antimalarial [23], anti-inflammatory [22,24], antiproliferative [25,26], anti-angiogenic [27], anti-tumour [28], anti-ageing properties [29], anti-arthritic [30], anti-Alzheimer’s [31,32], hypoglycaemic [33] and antiulcer [31,32]. These biological actions could be attributed to the presence of versatile bioactive phytochemical constituents, amongst which curcuminoids (1–6%) play an important role.

Curcuminoids are polyphenolic compounds consisting of curcumin and its derivatives (bisdemethoxycurcumin and demethoxycurcumin) [34,35], which have been widely explored over the last two decades for their multiple biological activities. Amongst all three, curcumin has been much explored for its cytotoxicity, mechanistic pathway and the enhancement of its pharmacokinetic as well as pharmacodynamic properties. However, the other two have still not received much attention [36]. Hence, this research aimed to provide a comparative study of the cytotoxic potential of different extracts of a *Curcuma longa* rhizome and curcuminoid mixture on cancer and healthy cell lines (i.e., DU-145, SCC-29B and Vero cells). The rationale behind the selection of the prostate cancer cell line (i.e., DU-145) was the GLOBOCAN world cancer data, which describe that prostate cancer is responsible for one-third of cancer cases in males, while the oral cancer cell line was selected based upon the Indian cancer data from 2020, released by IARC [7]. The third cell line, Vero cells, was chosen to compare the cytotoxic potential of these compounds on cancerous cells as well as healthy cells. This study also describes the isolation procedure of bisdemethoxycurcumin, its characterisation through different spectroscopic techniques and, for the first time, the cytotoxic potential on these three cell lines.

## 2. Experimental Section

### 2.1. Plant Material

The rhizomes of *Curcuma longa* were collected from Khari Baoli, Delhi, India, in July 2018 and authenticated by Sunita Garg, an emeritus scientist at CSIR-NISCAIR (Council of Scientific and Industrial Research, National Institute of Science Communication and Information Resources), Delhi, with the voucher no. NISCAIR/RMD/Consult/2018/3239-40. The required amount of authenticated plant rhizomes was purchased from the same place in Delhi and were cleaned, sliced and dried under the sun for seven days. After again drying the rhizomes, they were ground and sieved using sieve no. 60 [37] and stored in an air-tight and light-protected container.

### 2.2. Preparation of Plant Extracts

The coarsely ground *Curcuma longa* rhizomes were accurately weighed at 10 gm for each extraction using three different solvents (100 mL) of varying polarities: chloroform (CHCl_3_) (boiling point: 61.2 °C), ethanol (C_2_H_5_OH) (boiling point: 78.37 °C) and a hydroalcoholic solution (60 mL methanol and water 40 mL). The chloroform and ethanolic extractions were carried out using the Soxhlet apparatus for 6 h, while the hydroalcoholic extraction was conducted via a cold maceration process for five days. After extraction, the excess solvents were re-collected via reflux condensation and the obtained extracts were cooled (if hot), filtered and concentrated using the rotary evaporator.

### 2.3. Screening Test for Phytochemicals

All three extracts were qualitatively screened for the presence of constituents, such as saponins, coumarins and tannins, as these compounds contribute an important role in different biological activities including anti-inflammatory, pain-relieving, anticancer, antihypertensives and anxiolytic [38]. All these chemicals or reagents utilised for phytochemical tests were bought in an already prepared form. The test for different phytochemicals was as follows: 0.5 mL of all three extracts were taken in different test tubes, and 2 mL of dilute hydrochloric acid was added and filtered. The filtered extracts were treated with 1 mL of Dragendorff’s reagent to observe orange-red precipitation, which can be considered as a marker for alkaloids. For testing the presence of carbohydrates, the extract (2 mL) was treated with Molisch reagent (1 mL) and a few drops of concentrated sulphuric acid. The formation of a violet ring confirmed the presence of carbohydrates in the given samples. For testing the presence of glycosides, the available extracts were measured (2 mL), treated with chloroform (3 mL) and 10% ammonia solution. The development of a pink colour was ascertained due to the presence of glycosides.

For testing the presence of saponins, 20 mL water was added to all three samples (1 mL) kept in an individual graduated cylinder. The cylinder was shaken for 15 min to check for the presence of a foamy layer that only occurs if saponins are present. The presence of steroids was checked by adding 1 mL of each extract and 1 mL chloroform in each test tube, and a few drops of concentrated sulphuric acid was added to observe or any change in colour. If the solution turned red, then the test could be considered positive. A screening test for proteins was performed by adding an equal volume of biuret reagent solution and each extract (2 mL) to each test tube. The appearance of a bluish violet colour indicated the presence of proteins. The presence of terpenoids was checked by the formation of red brown colour at the interface when chloroform (2 mL) and concentrated sulphuric acid reacted with 0.5 mL of each extract. For determining the presence of flavonoids, a filter paper was dipped in the extract solution and ammonia was further added to the solution to observe the presence of a yellow colour. The Borntrager’s test was performed for checking the presence of anthraquinones by adding an equal amount of each extract (1 mL) and sulphuric acid to the test tube and then boiled and filtered. An equal amount of chloroform was added, and its layer was separated. To this formed layer, an equal amount of dilute ammonia solution was added. The presence of a pink to red colour indicates the test was positive for anthraquinones. A hydrochloric acid test was performed for checking for the presence of phlobatannins by adding a few drops of hydrochloric acid (2% *v*/*v*) into the extract (1 mL), and if a red colour precipitate appeared, phlobatannins were present.

Afterwards, a small quantity of extract (1 mL) was added to the water and then heated in a water bath. To this heated mixture, a few drops of 0.1% *w*/*v* ferric chloride solution was added; the appearance of a dark green colour indicated the presence of tannins [39].

## 3. Isolation of Curcuminoids

Accurately weighed 20 gm of coarsely powdered rhizomes were extracted with acetone solvent for 6 h using a Soxhlet Extractor and was filtered and concentrated using a rotary evaporator. The obtained extract was precipitated by adding petroleum ether, filtered and further dried by vacuum suction. The obtained curcuminoid mixture was further analysed using thin-layer chromatography (TLC) and a mixture of chloroform and methanol (9.5:0.5) as a solvent, and the spots that appeared were identified based on their Rf values [40]. The same procedure was also repeated in two other replicas for the isolation of bisdemethoxycurcumin.

### 3.1. HPLC Analysis of Curcuminoids

The analysis of curcuminoid was performed using a Perkin Elmer Series 200 Diode array detector (single-beam polychromators with a source of deuterium and tungsten-halogen, wavelength ranging from 190 to 700 nm, 512 diodes, sensitivity ranging from 0.0001 to 2.0 AUFS); a UV detector and a fluorescence detector that can work on isocratic and gradient modes. In addition, a Series 200 vacuum degasser and C18 column (250 × 4.6 mm i.d.; 5 μm; Alltech Associates, Inc., Deerfield, IL, USA) fitted in the instrument. The sample analysis was carried out in an isocratic system with a 1.0 mL/min sample flow rate and 35 °C temperature. The mobile phase employed was a mixture of acetonitrile: water (60:40) (HPLC grade) and 2% acetic acid (40:60, *v*/*v*) (HPLC grade) at a detection wavelength of 425 nm.

The sample was weighed (100 mg) and sonicated in acetonitrile (100 mL) for 10 min. Then, 1 mL of this solution was taken and diluted in 2.5 mL of solvent to obtain a concentration of 400 µg/mL, which was further diluted ten times to get 40 µg/mL. Next, the solution (10 μL) was filtered through a 0.50 μm nylon membrane and finally injected into the column for performing the HPLC.

### 3.2. Isolation of Bisdemethoxycurcumin

The bisdemethoxycurcumin was separated from the curcuminoid mixture by the column chromatography technique by using a mixture of crude curcuminoid and silica gel (60–120 mesh) (5:8) in the C18 column having dimension 250 × 4.6 mm i.d.; 5 μm (Alltech Associates, Inc., Deerfield, IL, USA) already packed with silica gel slurry. Then, the sample elution started with non-polar solvent chloroform followed by increasing polarity by adding a mixture of chloroform and methanol in a ratio of 95:5. As a result, all the obtained fractions were analysed by TLC silica gel 60 F254 plate, and the spots appeared as a yellowish-orange colour. After analysing all the fractions, the fractions with similar Rf values were pooled to form a single fraction and concentrated by removing the excess organic solvent.

The obtained crude bisdemethoxycurcumin was purified by solubilising in solvent methanol and then heated. Upon complete solubilisation, chloroform was further added until the ratio of methanol: chloroform was 5:2. This mixture was kept undisturbed overnight at 5 °C, and the purified compound was filtered and lastly precipitated with petroleum ether. HPLC and the melting point analysis of purified compounds were further performed to determine their level of purity [40].

### 3.3. Melting Point Determination

The melting point of the obtained crystals was determined using a WEB CON Instruments (Varanasi, Uttar Pradesh) melting point apparatus.

### 3.4. HPLC Analysis of Bisdemethoxycurcumin

The obtained crystals were dissolved in ethanol and filtered through a 0.5 µm nylon membrane for injecting (20 µL) into the HPLC column. The HPLC column employed for the analysis was C18 of Alltech Associates, Inc. (Deerfield, IL, USA), fitted in a Shimadzu LC-Prominence 20AT instrument. This analysis utilised a methanol: water (50:50) (HPLC grade) as the mobile phase at a flow rate of 1.0 mL/min and temperature of 35 °C, and the spectral peaks were detected by a diode array detector at a wavelength of 425 nm.

### 3.5. IR (Infrared) Spectroscopy

For confirming the structure of the isolated compound, infrared spectroscopy was performed that determined the presence of a functional group in an isolated compound. The sample was weighed (0.1 mg) and mixed with 100 mg potassium bromide (KBr) for performing the same. This mixture was pressed to form a pellet that was analysed on a Perkin Elmer Spectrum GX Range 10,000–370 cm^−1^ having an ATR accessory for reflectance measurement and IR Quant software [41].

### 3.6. NMR (Nuclear Magnetic Resonance)

The ^1^H and ^13^C NMR spectra of the isolated sample were determined in 400 MHz Supercon NMR Spectrometer from Bruker, West Germany, with the multinuclear probe, commonly meant for the study of ^1^H, ^13^C, ^31^P, ^11^B, and 2D experiments such as DEPT (distortionless enhancement by polarisation transfer), which gives the CH, CH_2_, and CH_3_ carbons. The sample to be analysed was dissolved in D_2_O and operated at 200 and 50 MHz, respectively. The obtained proton and carbon spectra matched with the standard structure of the compound.

### 3.7. Mass Spectrometry

The sample was analysed in a TSQ Quantum Access MAX Triple Quadruples LCMS2010A, the advanced version for mass studies from Shimadzu, Japan. This instrument can study compounds with molecular weights up to 2000 and fitted with atmospheric pressure chemical ionisation (APCI) and electron spray ionisation (ESI) probes useful for studying non-polar and polar compounds. The following parameters were set for performing the analysis such as curtain gas 10, gas 1(20), gas 2 (0), needle voltage 5000 V, and declustering potential 100 V. The sample to be analysed was dissolved in water, and 5 μL of the sample was injected into LC-MS. The scan operated in both the positive and negative ion modes with precursor ion mass scans ranging from 50 to 1050 daltons.

## 4. Cancer Cell Lines

The current investigation included DU-145 (human prostate cancer cell line), SCC-29B (human oral carcinoma cell line) and a normal Vero cell line (monkey normal kidney cell line). These cell lines were procured from American Type Culture Collection (ATCC) and were cultured in Dulbecco’s modified Eagle’s medium (DMEM), supplemented with foetal bovine serum (10% FBS) (inactivated), penicillin (100 IU/mL) and streptomycin (100 µg/mL) with 5% carbon dioxide at a temperature of 37 °C. The cells were dissociated using 0.2% trypsin, 0.02% EDTA and 0.05% glucose in phosphate buffer solution. The cell viability was analysed and, furthermore, 50,000 cells/well were seeded in a 96-well plate and incubated for one complete day (37 °C and 5% CO_2_).

## 5. Cytotoxicity Assay

The in vitro cytotoxicity assay of the plant extracts, curcuminoids and bisdemethoxycurcumin, was performed using the 3-(4,5-dimethylthiazol-2-yl)-2,5-diphenyltetrazolium (MTT) assay. For performing the assay, 100 µL of the diluted cell suspension (50,000 cells/well) was added to each 96-well microtiter plate. After one complete day (24 h), the formation of a partial monolayer was observed and the remaining supernatant was decanted. The preformed monolayer was washed once with the medium. Approximately 100 µL of the test samples were added to the partial monolayer in microtiter plates and the plates were incubated (37 °C; 5% CO_2_; 24 h). After 24 h of incubation, 100 µL of MTT (5 mg/10 mL of MTT in PBS) was added to each well, and the plates were again incubated for 4 h at the same conditions. The supernatant was decanted again, and 100 µL of DMSO was added and the mixture was shaken gently to dissolve the formed formazan. The mixture’s absorbance was measured at a wavelength of 590 nm.

The assay employed the calculation of the IC_50_ value, which can be calculated using the following formula:

% Inhibition (IC_50_) = ((OD of Control − OD of sample)/OD of Control) × 100.

## 6. Preparation of Test Solutions

Standard: doxorubicin

**Figure 1:**
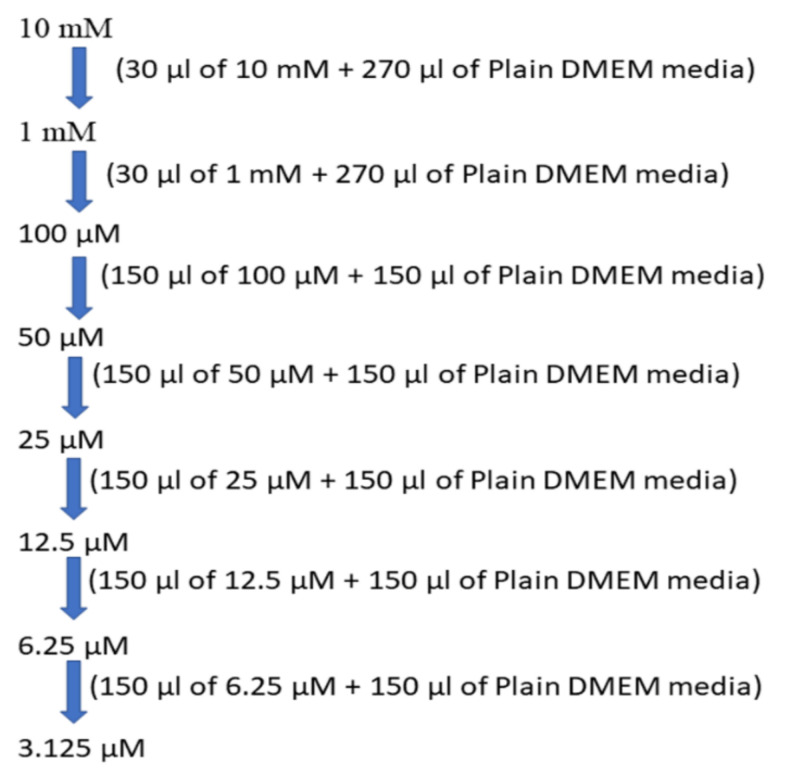
Concentrations of the standard drug solution.

For cytotoxicity studies, the prepared dilutions were 3.125, 6.25, 12.5, 25, 50 and 100 µM, which is depicted in Figure 1.

Standard Concentrations:

### Test Sample Preparation

**Figure 2:**
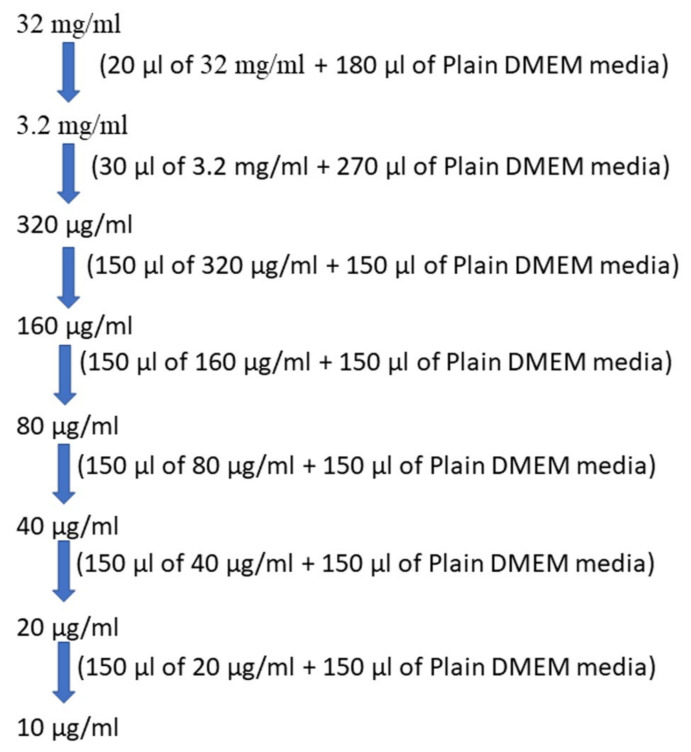
Concentrations of test drug solution.

For cytotoxicity studies, 32 mg/mL stock solution was prepared using DMSO for BD. Then, the serial two-fold dilutions were prepared from 320 to 10 µg/mL using DMEM plain media for treatment. The preparation of the different sample concentrations 10, 20, 40, and 80 µg/mL is depicted in Figure 2.

## 7. Statistical Analysis

The cytotoxicity results were statistically analysed using one-way ANOVA. All the IC_50_ values of the cytotoxic studies, represented as the mean ± standard error mean (SEM), were statistically analysed by comparing the cytotoxicity of all extracts with the standard drug doxorubicin using a one-way ANOVA test. The calculated value of the f-ratio was 1.1873, which is less than the table value 4.26 at a 5% level of significance (*p*-value 0.05) with a degree of freedom (d.o.f.) of 3 and 8. As 1.1873 is less than 4.26, this proves the significance of the obtained results.

## 8. Results and Discussion

### 8.1. Percentage Yield of Extraction

Plants contain multiple phytoconstituents, such as flavonoids, saponins, tannins and glycosides, the properties of which differ entirely from each other on one or another aspect. Hence, it is quite impossible to extract every phytochemical constituent via a single solvent. Therefore, the polarity-based variation in the solvent system was applied for the selection of solvent system, i.e., from non-polar (chloroform) to highly polar (ethanolic). The constituents will be extracted in their respective solvents based on the nature of the phytoconstituents present. The amount of obtained extract and its percentage yield is reported in Table 1 using the following formula:

% Yield of extract = weight of crude × 100/weight of the sample

Based on the results of the extractive yield, it can be concluded that ethanol extract had a greater number of phytochemical constituents and showed greater biological activity.

### 8.2. Phytochemical Screening

The presence of numerous phytochemical constituents in plants provide versatile biological properties for multipurpose utilisation such as treating ailments, cosmetics, flavours and fragrance. A qualitative analysis was performed on all three turmeric extracts via different screening tests, and the results are recorded in Table 2. The obtained results showed that ethanolic (EC1) and hydroalcoholic extract (CMW) contained more phytochemical constituents rather than chloroform extract. The chloroform extracts (CC1) lacked the presence of proteins, terpenoids, flavonoids and tannins, while the hydroalcoholic extract did not contain phlobotannins. Amongst the three, the ethanolic extract was found to be rich in a maximum number of phytochemical constituents that can form the basis for its much stronger potential. Moreover, it can also be concluded that the greater the polarity a solvent can extract, the more versatile phytochemical constituents for greater biological utilities. Based upon the preliminary literature, it was found that the ethanolic extract contained the highest fraction of curcumin and other phenolics, which justifies its antioxidant property and use in traditional medicine [42].

### 8.3. Isolation of Curcuminoids

**Figure 3:**
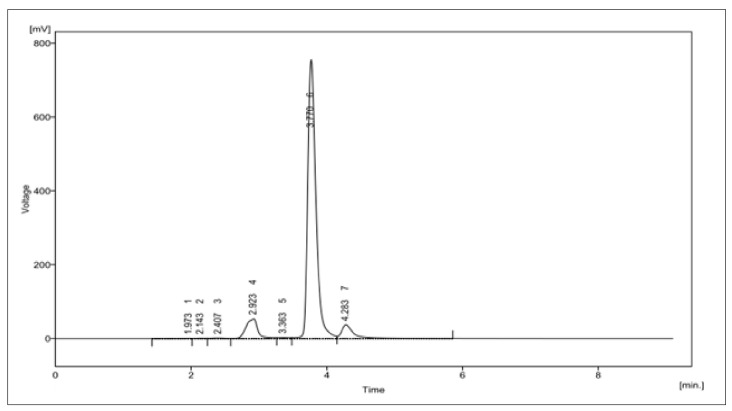
HPLC of curcuminoids.

Curcuminoid contains a mixture of three compounds: curcumin, demethoxycurcumin and bisdemethoxycurcumin; each of them possesses immense biological potential. These curcuminoids were extracted using acetone as a solvent in a raw form, as it yields the maximum extractive value from the coarsely powdered sample. The obtained yield was purified by adding petroleum ether, which is further filtered and dried under vacuum suction to obtain the compound in pure form. The obtained yield is shown in Table 3:

The obtained curcuminoid mixture was applied as a spot on the silica gel F254 coated plates, using chloroform: methanol (9.5:0.5) as the developing solvent system. With the migration of the solvent mixture, three spots were identified based on their Rf values which were 0.73, 0.52 and 0.3 for curcumin, demethoxycurcumin and bisdemethoxycurcumin similar to authentic standards as reported in the literature [43]. The same mixture was also analysed using the HPLC technique, and its spectra are shown in Figure 3.

### 8.4. HPLC Analysis of Curcuminoids

The HPLC analysis of curcuminoids showed three major peaks, expressing the presence of three compounds, namely, bisdemethoxycurcumin, curcumin, and demethoxycurcumin at Rt values of 2.923, 3.770 and 4.283 min. The area percentage enclosed by these peaks were 8.6%, 84.0% and 6.3%. Hence, this HPLC in Figure 3 further confirms that the isolated curcuminoids were found to be a mixture of only three major compounds. The details of this HPLC analysis are expressed in Table S1 of the Supplementary Materials.

### 8.5. Isolation of Bisdemethoxycurcumin

For isolating the bisdemethoxycurcumin, the acetone extracted curcuminoid mixture was subjected to column chromatography. The elution was done using chloroform followed by chloroform: methanol with increasing polarity. All the obtained fractions were spotted on TLC, and fractions that showed the same pattern in TLC were pooled and concentrated. The pooled fractions showed the different curcuminoid compositions such as curcumin; curcumin and demethoxycurcumin; demethoxycurcumin, demethoxycurcumin and bisdemethoxycurcumin; Bisdemethoxycurcumin. The last pooled fraction that contained only bisdemethoxycurcumin was retained, and the remaining were discarded. Then, the required pooled fraction was concentrated and purified by the chloroform and methanol solvent mixture to obtain the exact practical yield of the yellowish-orange-coloured crystals. The obtained final yield was 368.5 mg or 7.36% *w*/*w*, and its details are expressed in  Supplementary Materials Table S2.

### 8.6. Melting Point

The melting point of the obtained crystals was estimated to be 226 °C, while the standard should lie between 226 and 231 °C, thereby confirming that the crystals could be of bisdemethoxycurcumin.

### 8.7. Characterisation

#### 8.7.1. HPLC Analysis of Bisdemethoxycurcumin

**Figure 4:**
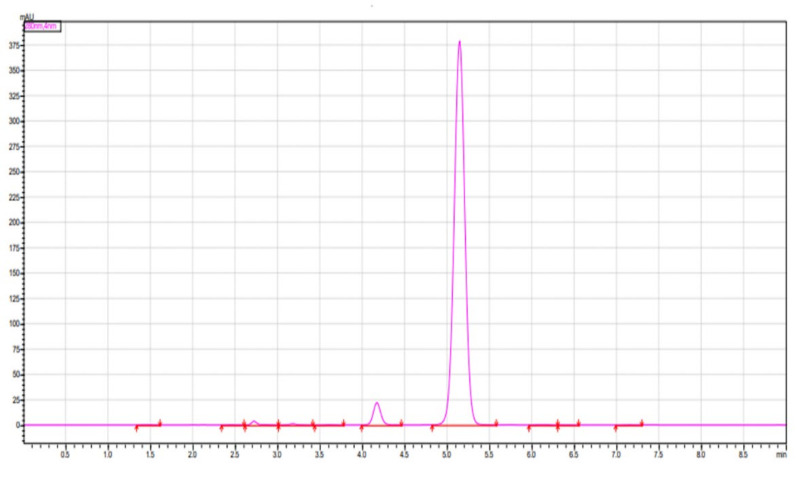
HPLC of isolated bisdemethoxycurcumin.

The HPLC analysed the purity profile of isolated bisdemethoxycurcumin, shown in Figure 4, depicting single BD peaks at a retention time of 4.978 min with an area percentage of 98.120. These purified crystals were further characterised for their structure by various spectroscopic techniques and for biological potential via cytotoxicity assay. The details of this HPLC analysis are expressed in Table S3 of the Supplementary Materials.

#### 8.7.2. FT-IR Spectra

**Figure 5:**
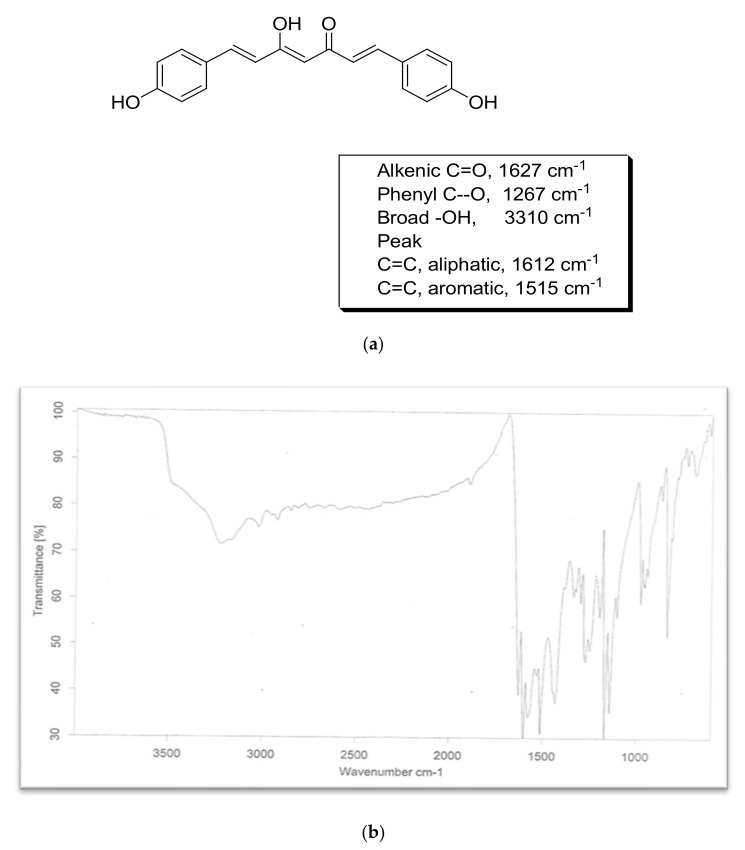
FTIR of bisdemethoxycurcumin. (**a**) Structure & chracteristic peaks of bisdemethoxycurcumin; (**b**) FTIR spectra of bisdemethoxycurcumin.

The infrared spectra of the sample were found to be in conformity with the already reported structures of bisdemethoxycurcumin. The obtained spectra did not express any peak in the methoxy region, which differentiated the obtained spectra from the other two curcuminoids. The FTIR spectra of bisdemethoxycurcumin are shown in Figure 5.

#### 8.7.3. Nuclear Magnetic Resonance

**Figure 6:**
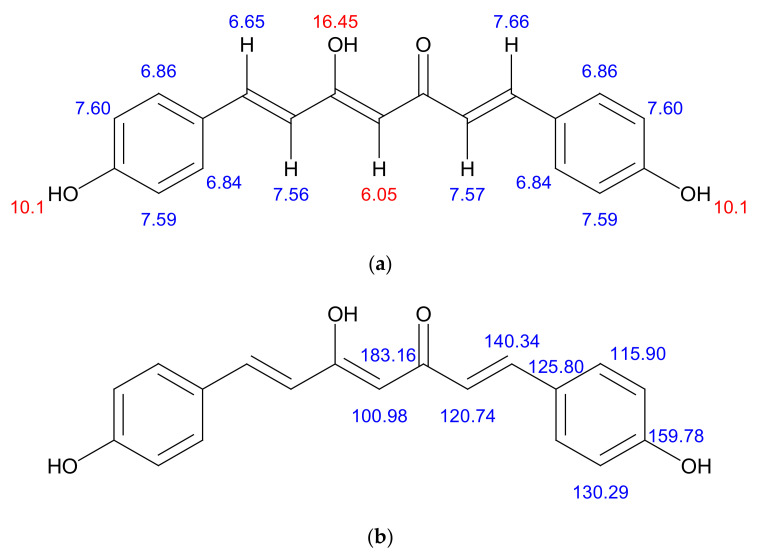
(**a**) structural characterisation of protons in the bisdemethoxycurcumin. ^1^H NMR (d6–DMSO, 400 MHz, δ, TMS = 0): 16.45 (^1^H, s, –OH), 10.10 (2H, s, Ar-OH), 7.57–7.60 (6H, m, ArH, –CH–), 6.69–6.86 (6H, m, ArH, –CH–), 6.05(^1^H, s, –C–H); (**b**) structural characterisation of carbons in the bisdemethoxycurcumin. ^13^C NMR (d6– DMSO, 400 MHz, δ, TMS = 0): 100.98, 115.90, 120.74, 125.80, 130.29, 140.34, 159.78, 183.16.

The obtained compound was further identified by the complete proton and carbon NMR spectra in D_2_O and confirmed as bisdemethoxycurcumin. The details of the spectra are given below (Figure 6a,b). ^1^H NMR (d6-D_2_O, 500 MHz, δ, TMS = 0):

*Proton NMR*


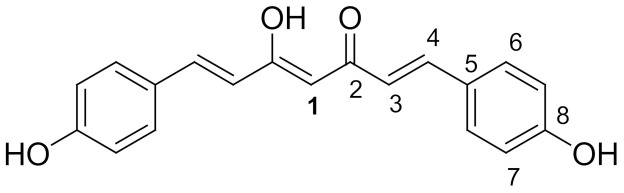


#### 8.7.4. Mass Spectrum

**Figure 7:**
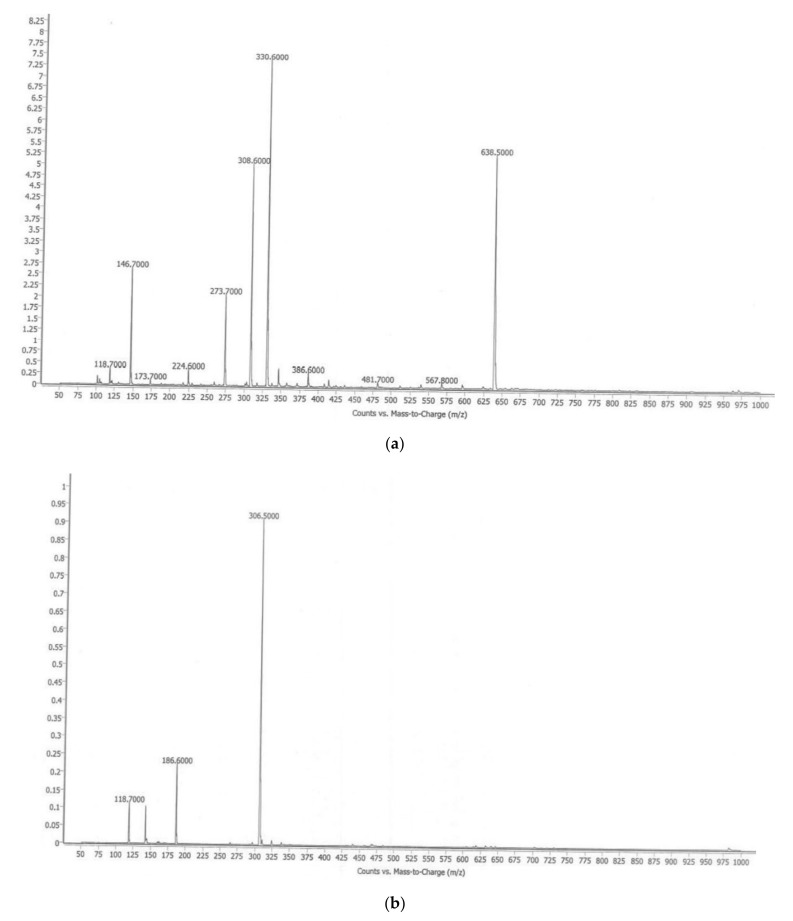
(**a**). LC-MS of BD (positive ionisation); (**b**). LC-MS of BS (negative ionisation).

The LC-MS spectra of the obtained sample exhibited a peak at mass *m*/*z*: 308.6 (M+ +1) positive ionisation and 306.5 (M+ −1) negative ionisation, which was found to match the reported molecular weight, confirming that the obtained sample was bisdemethoxycurcumin. The positive and negative ionisation peaks are shown in Figure 7a,b.

### 8.8. Cytotoxicity

**Figure 8:**
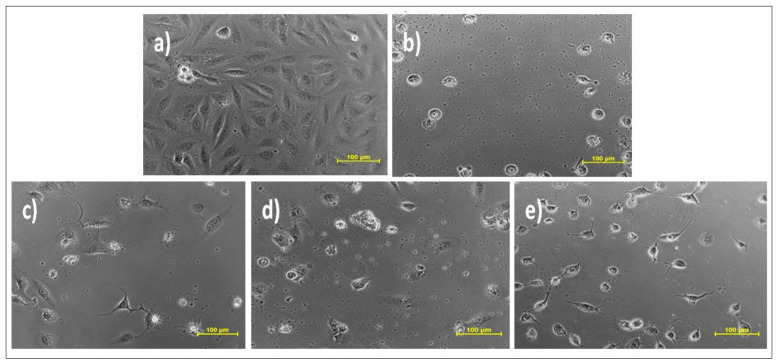
Effect of different samples on oral cancer cell line SCC-29B.Samples: (**a**) DMSO (dimethylsulfoxide); (**b**) doxorubicin (Dox); (**c**) ethanolic extract (EC1); (**d**) curcuminoid (C3); (**e**) bisdemethoxycurcumin (BD).

**Figure 9:**
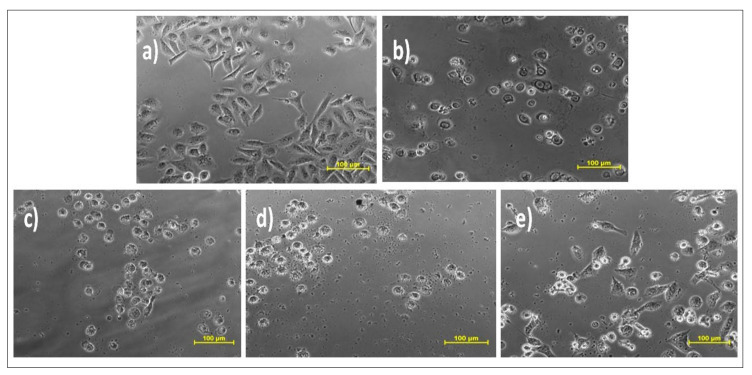
Effect of different samples on prostate cancer cell line DU-145.Samples: (**a**) DMSO; (**b**) doxorubicin (Dox); (**c**) ethanolic extract (EC1); (**d**) curcuminoid (C3); (**e**) bisdemethoxycurcumin (BD).

**Figure 10:**
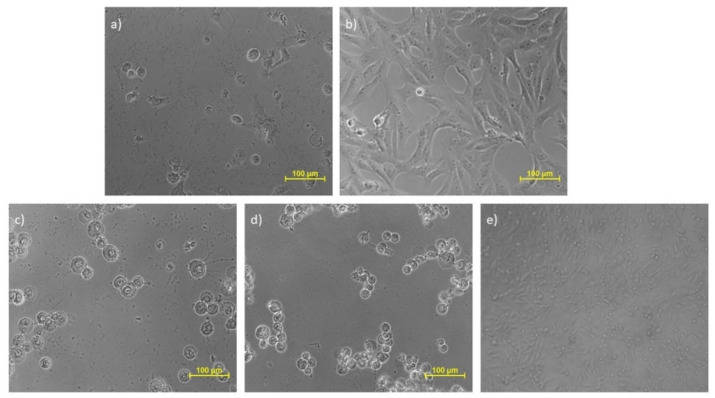
Effect of different samples on the Vero cell line. Samples: (**a**) DMSO; (**b**) doxorubicin (Dox); (**c**) ethanolic extract (EC1); (**d**) curcuminoid (C3); (**e**) bisdemethoxycurcumin (BD).

The cytotoxic potential of all the three plant extracts (EC1, CMW and CC1), curcuminoids (C3) and isolated compound bisdemethoxycurcumin (BD) was explored by MTT assay on three different cell lines that included prostate cancer (DU-145), oral cancer (SCC-29B) and healthy (Vero) cell lines. The addition of one healthy cell line was conducted to determine and compare the cytotoxic effect of the test samples (extract samples and isolated compounds) and the standard drug (doxorubicin) on normal and cancerous cells using their IC_50_ values. Amongst all the three extracts, the IC_50_ value of the ethanolic extract was found to be comparatively lower in both oral as well as prostate, proving its higher potency. However, its sensitivity was more against oral cancer, while the doxorubicin was highly potent at all doses in both types of cancer. The isolated compound bisdemethoxycurcumin also exhibited more efficacy in prostate cancer than oral cancer and was also less toxic to healthy cell lines. Considering the normal Vero cell line, ethanolic extract and bisdemethoxycurcumin depicted toxicity at quite higher doses in comparison to all others. The calculated IC_50_ values and their standard error means (SEM) is depicted in Table 4. The images of the oral and prostate cancer cell lines treated with vehicle control, ethanolic extract, curcuminoid, bisdemethoxycurcumin and the standard drug are depicted in Figure 8, Figure 9 and Figure 10.

The results show that the ethanolic extract of *C. longa* showed comparatively more cytotoxic potential in comparison to the non-polar and aqueous mixtures. The more cytotoxic potency of the ethanolic extract could be attributed to the presence of more amount of phenolics including all three curcuminoids [38]. Our results were also found in coordination with a previous study by Unnikrishnan et al. (1990) and Irshad et al. (2017) that stated that less cytotoxicity could be attributed to low solubility and stability of biological potent molecules in non-polar or aqueous mixtures [43,44].

Teiten et al. also reported the cytotoxic activity of curcumin on prostate cancer by interfering with the cells’ proliferation and metastasis by downregulating the androgen receptor and epidermal growth factor receptor and inducing cell cycle arrest. It induces the ability of pro-apoptotic proteins and downregulate the anti-apoptotic counterparts [45]. Even demethoxycurcumin was found to be effective in prostate cancer. It acts by AMPK-induced downregulation of HSP70 and EGFR [46]. Even the phthalimide derivatives prepared from curcumin also showed great cytotoxicity in prostate cancer that acts by altering the expression of key genes controlling cell proliferation, such as Cylins D1, B1 and B2 and apoptosis, among the Puma, Noxa and Bcl-2 family members [47]. The activity of both the curcuminoids have been explored, and this article also showed that bisdemethoxycurcumin was also found to be effective in treating prostate cancer. The use of curcumin was also explored in oral cancer in the form of 0.1% curcumin mouthwash and found to significantly delay the onset of RIOM (Clinical trial registration no. CTRI/2018/04/013362) [48].

## 9. Conclusions

The results of a comparative phytochemical screening and the cytotoxic potential of the *Curcuma longa* extracts showed that the more polar solvents exhibited (ethanolic extract) the presence of major phytochemical constituents as well as significant cytotoxic potential. In addition, they were comparatively safer for the non-cancerous cell lines. This indicates that the ethanolic extract possesses selective cytotoxicity for the cancerous cell lines, making the non-cancerous cell line safer from its cytotoxic effects. Besides these extracts, the isolated curcuminoid also showed comparable cytotoxic potential to that of ethanolic extract in SCC-29B and DU-145 but unfortunately were found to be cytotoxic for the Vero cell lines too. Considering bisdemethoxycurcumin, it also showed good cytotoxic potential and was found to be quite safe in the Vero cell lines when compared to the standard drug doxorubicin.

## 10. Future Prospects

Looking at the results of the comparative cytotoxic potential of extracts, the ethanolic extract has shown a greater cytotoxic potential with a quite high specificity for the cancer cell line. Hence, this extract can be explored further in future to determine the potent constituent that could be responsible for this specific cytotoxic effect. These studies, which were performed in vitro on the cell lines, can form the predictive basis for performing the studies in vivo and can be taken up in the future for obtaining more reliable and standardised results. Moreover, bisdemethoxycurcumin showed good cytotoxic potential but was not comparable to curcuminoids. The advantage of considering bisdemethoxycurcumin in the future is its lower cytotoxicity for healthy cell lines and comparatively greater stability among all the three curcuminoids.

## Figures and Tables

**Table 1:** The percentage yield of crude extracts of *Curcuma longa* rhizomes.

Sample Number	Solvents	Extractive Value	% Yield (*w*/*w*) ^a^
1	Chloroform	249 mg	2.49%
2	Ethanol	1104 mg	11.04%
3	Hydro-alcoholic (60:40)	348 mg	3.48%

^a^ Dried sample weight was 10 gm.

**Table 2:** Phytochemical screening of *Curcuma longa* rhizome extract.

Test	Ethanolic Extract (EC1) ^a^	Chloroform Extract (CC1) ^a^^,^^b^	Hydroalcoholic Extract (CMW) ^a,b^
Alkaloids	+	+	+
Carbohydrates	+	+	+
Glycosides	+	+	+
Saponins	+	+	+
Steroids	+	+	+
Proteins	+	−	+
Terpenoids	+	−	+
Flavonoids	+	-	+
Anthraquinones	+	+	+
Phlobotannins	+	+	−
Tannins	+	−	+

Where ^a^ (+) indicates the presence of a phytochemical constituent and ^b^ (−) indicates the absence of a phytochemical constituent.

**Table 3:** The percentage yield of curcuminoid from *Curcuma longa* rhizomes.

Sample Number	Solvents	Quantity Taken	Extractive Value	% Yield (*w*/*w*)
1	Acetone	20 gm	3.2 gm	16.0%
2	Acetone (for the other 2 replicas)	20 × 2= 40 gm	3.2 × 2 = 6.4 gm	16.0%
Total yield = 3.2 + 6.4 = 9.6 gm for 60 gm of rhizomes				

**Table 4:** Cytotoxicity profile of *Curcuma longa* extract and its isolated compounds.

Cell Type	Cell Line	IC_50_ (μg/mL)					
		EC1 ^b^	CMW ^b^	CC1 ^b^	C3 ^b^	BD ^b^	Dox ^a,b^
Prostate Cancer	DU-145	19.88 ± 0.5	53.98 ± 0.27	1365.47 ± 0.36	17.82 ± 0.6	93.28 ± 0.5	19.5 ± 0.5
Oral Cancer	SCC-29B	11.27 ± 0.37	32.22 ± 0.51	426.896 ± 0.5	16.79 ± 0.56	106.91 ± 0.45	17.53 ± 0.5
Normal Kidney cell line (healthy cells)	Vero cells	525 ± 0.5	16.80 ± 0.66	−107.915 ± 0.8	23.9 ± 0.45	202.1 ± 0.5	53.39 ± 0.5

^a^ Dox = doxorubicin (standard drug); ^b^ SEM = ±standard error mean.

## Data Availability

Not applicable.

## Supplementary Materials

The following are available online, Table S1: HPLC analysis of Curcuminoids, Table S2: The final yield of isolated Bisdemethoxycurcumin, Table S3: HPLC analysis of Bisdemethoxycurcumin.
