# Supplementary material for: In Vitro Phytochemical Screening, Cytotoxicity Studies of Curcuma longa Extracts with Isolation and Characterisation of Their Isolated Compounds
Source: Molecules. 2021 Dec 11;26(24):7509. doi: 10.3390/molecules26247509 (PMC8705887; doi:10.3390/molecules26247509)
Supplement: Supplementary file 1 [file molecules-26-07509-s001.zip › molecules-1489132-supplementary.pdf]

Supplementary data

Table S1: HPLC analysis of Curcuminoids

Peak	Reten. Time [min.]	Area	Height [mV]	Area [%]	Height [%]
1	1.973	11.920	0.842	0.2	0.1
2	2.143	11.821	1.053	0.2	0.1
3	2.407	22.049	1.503	0.3	0.2
4	2.923	646.001	53.343	8.6	6.3
5	3.363	32.571	2.637	0.4	0.3
6	3.770	6277.502	754.627	84.0	88.7
7	4.283	471.469	37.088	6.3	4.4
	Total	7473.333	851.092	100.0	100.0

Table S2: The final yield of isolated Bisdemethoxycurcumin

S. No.	Composition	Weight of extract[a]	Percentage (%) yield[a]
1	Bisdemethoxycurcumin (BD)	368.5 mg	7.36%

Where, [a] weight of Curcuminoids is 5 gm.

Table S3: HPLC analysis of Bisdemethoxycurcumin

Peak	Ret. Time	Area	Height	Conc. (mg/L)
1	4.169	241710	50002	1.820
2	4.978	1223921	38082	98.120
Total		1465631	137856	100
